# Risk Factors for Adult Axial Length Elongation: A 5-Year Population-Based Cohort Study

**DOI:** 10.1016/j.xops.2025.101011

**Published:** 2025-11-17

**Authors:** Taiga Inooka, Yuki Kimura, Shota Fujikawa, Sayuri Yasuda, Taro Kominami, Tetsuhito Kojima, Shinji Ueno, Yasuki Ito, Koji M. Nishiguchi, Kenya Yuki

**Affiliations:** 1 Department of Ophthalmology, Nagoya University Graduate School of Medicine, Nagoya, Japan; 2 Aichi Health Promotion Foundation, Nagoya, Japan; 3 Department of Ophthalmology, Hirosaki University Graduate School of Medicine, Hirosaki, Japan; 4 Department of Ophthalmology, Fujita Health University School of Medicine, Toyoake, Japan

**Keywords:** Axial length elongation, Adult myopia, Interocular difference, Longitudinal cohort, Risk profiling

## Abstract

**Purpose:**

Adult axial length (AL) elongation in adults is associated with pathologic outcomes; however, population-based longitudinal evidence remains limited, and pragmatic risk profiling is unclear. We aimed to quantify the prevalence and annual rate of AL elongation in adults and identify independent determinants in a population-based health-check cohort.

**Design:**

Retrospective, single-center cohort study.

**Subjects:**

A total of 4016 adults aged 22.4 to 93.0 years (8032 eyes; 21 421 visits) undergoing a Japanese health-check program, with a median follow-up of 5.31 years.

**Methods:**

For each eye, the annual AL change (mm/year) was estimated as the within-eye linear-regression slope and classified as severe (≥0.10), moderate (≥0.05 to <0.10), mild (≥0.00 to <0.05), or nil (<0). Associations were evaluated using a class-weighted proportional-odds ordinal logistic model. Pair-level change-point analysis modeled which eye elongated faster.

**Main Outcome Measures:**

Proportion of eyes by annual AL elongation severity, adjusted odds ratios (ORs) for determinants of more-severe elongation, and the baseline-AL intersection at which the longer eye becomes more likely to elongate faster.

**Results:**

Nil and mild accounted for 98.6% of eyes; moderate and severe were uncommon (1.3% and 0.2%). Independent determinants of greater severity included longer baseline AL (OR, 1.34 per 1 mm), larger interocular difference in AL (OR, 7.18 per 1 mm), myopic maculopathy including tessellated fundus (OR, 2.79), and female sex (OR, 3.05). Change-point analysis identified an intersection near 28.56 mm in the longer eye: below this value, the estimated probability that it would elongate faster was approximately 0.50 (no consistent lateral preference), whereas above it the probability exceeded 0.50 with wide uncertainty at high baseline AL values.

**Conclusions:**

Adult AL elongation is uncommon and slow; risk is concentrated in eyes with longer AL, greater axial asymmetry, myopic maculopathy, and in female adults. These readily measured features can inform follow-up decisions in health-check settings; pair-level estimates around 28.6 mm may help prioritize eyes for follow-up but should be interpreted cautiously.

**Financial Disclosure(s):**

Proprietary or commercial disclosure may be found in the Footnotes and Disclosures at the end of this article.

Myopia is increasing globally, with the prevalence of high myopia projected to increase from 2.7% in 2020 to 9.8% by 2050,^1^^,^^2^ posing a substantial clinical and public health challenge.^2^ Although childhood progression predominates, axial length (AL) can continue to elongate in adulthood.^3^ Annual AL elongation ≥0.10 mm/year is associated with an increased risk of myopic macular degeneration, rhegmatogenous retinal detachment, cataract, and glaucoma.^3^, ^4^, ^5^

Recent hospital-based studies indicate that sustained AL elongation in adults occurs not only in pathologic myopia but also in nonpathologic high myopia,^4^^,^^6^^,^^7^ with longer baseline AL, female sex, younger age, and greater axial asymmetry implicated as potential risk factors.^7^ However, because the evidence largely comes from hospital-based cohorts and is prone to selection bias, whether the same tendencies hold in the general adult population across the spectrum from prepathologic to pathologic stages is uncertain.

Given the association between adult AL elongation and sight-threatening outcomes, identifying at-risk individuals in community settings is clinically important. To enable such pragmatic risk profiling, population-based longitudinal data are needed, together with analyses that appropriately account for intereye correlation and marked outcome imbalance. Accordingly, we analyzed a Japanese adult health-check cohort comprising 4016 participants with a median follow-up of 5.31 years, estimating per-eye annual AL change from ≥3 visits. We fitted a class-weighted ordinal logistic model with participant-clustered standard errors to identify determinants of more-severe adult AL elongation to help identify higher-risk eyes. Our primary objectives were to describe the distribution of adult AL elongation and identify factors associated with greater progression beyond high myopia.

## Methods

### Study Design and Ethics

This single-center, retrospective, longitudinal cohort study adhered to the tenets of the Declaration of Helsinki and was approved by the Institutional Review Board of Nagoya University Graduate School of Medicine (approval number 2025-0237 37403). Informed consent was obtained using an opt-out approach, and all data were anonymized before analysis.

### Participants and Setting

Participants were Japanese adults who underwent general health and ophthalmic check-ups at the Aichi Health Promotion Foundation between April 3, 2018, and March 31, 2025, as part of a population-based health-check program that provides general community health check-ups and workplace-based screenings for adults. Inclusion criteria were as follows: (1) age ≥20 years; (2) ≥3 visits; and (3) follow-up duration ≥20 months. Exclusion criteria were as follows: (1) a history of intraocular surgery other than cataract surgery (including vitrectomy, corneal surgery, laser in situ keratomileusis, retinal laser treatment/photocoagulation, or procedures after penetrating or open-globe ocular trauma); (2) conditions that could affect AL measurement as identified via questionnaire (lens dislocation, acute angle-closure, corneal laceration, uveitis, or vitreous hemorrhage); and (3) missing essential data (including insufficient data for either eye). History of cataract surgery was recorded as a self-reported variable.

All participants underwent comprehensive ophthalmic examinations, anthropometric measurements, blood tests, and a clinical interview on the same day. Corrected visual acuity was measured with an automated vision tester (CA-1000, Tomey). Refractive error was measured with an auto Ref/keratometer (RK-F2, Canon) without cycloplegia. Axial length was measured as the distance from the anterior corneal surface to the retinal pigment epithelium with a partial coherence interferometry device (OA-2000, Tomey). Intraocular pressure was measured with a noncontact tonometer (TX-20P, Canon). Color fundus photographs were obtained with a retinal camera (CR-2 AF, Canon) and myopic maculopathy was classified according to the Meta-Analysis for Pathologic Myopia (META-PM) category.^8^ For biochemical and hematologic assessments, fasting venous blood was drawn to measure lipids, indices of glucose metabolism, renal and hepatic function, and complete blood count. For anthropometry, height, weight, body mass index, blood pressure, and pulse rate were recorded. The questionnaire captured medical history, ocular surgical history (including the presence or absence of cataract surgery), lifestyle factors (alcohol consumption, smoking, and exercise), medication use (antihypertensives, antidiabetic agents, and lipid-lowering agents), and glaucoma treatment. Data quality control was independently verified by 2 ophthalmologists (T.I. and Y.K.).

### Variable Definitions

The terms “men” and “women” are used only when reporting on findings from previously published studies, in accordance with the terminology used in those original works. All analyses in the present study use biological sex (male/female). Spherical equivalent refraction (SER) was calculated as sphere + 0.5 × cylinder. Interocular difference in AL was defined as the absolute difference (mm) between right- and left-eye AL measured at the same visit. Corrected visual acuity recorded in decimal notation was converted to logarithm of the minimum angle of resolution units. For Meta-Analysis for Pathologic Myopia (META-PM) classification, the full scale (0–4) was considered; given the pronounced class imbalance with META-PM = 0 being overwhelmingly frequent, we used a binary indicator of META-PM ≥1 (vs. 0) in the proportional-odds analysis. Medication use was binarized as presence or absence of antihypertensives, antidiabetic agents, and lipid-lowering agents; history of glaucoma treatment was also binarized as present or absent. Habitual exercise was defined as engaging in exercise for >30 minutes per session at least twice per week for ≥1 year; habitual walking was defined as walking or an equivalent activity for ≥1 hour per day.

### Primary Outcome

The primary outcome was AL elongation (mm/year). For each eye, we fitted a univariable linear regression of AL against time (years) and defined the slope (β_1_) as the annual elongation rate. After a prior report,^7^ the estimated rate was categorized into the following 4 severity categories: (1) severe, ≥0.10 mm/year; (2) moderate, ≥0.05 to <0.10 mm/year; (3) mild, ≥0.00 to <0.05 mm/year; and (4) nil, <0 mm/year. These ordered categories served as the dependent variable for risk factor analyses.

### Statistical Analyses

All analyses were performed with Python version 3.6.7 (https://www.python.org/downloads/release/python-367/) and R version 4.2.2 (https://www.r-project.org/). Individuals with missing essential data were excluded as described above, and complete-case analyses were conducted. Continuous variables were summarized as median (interquartile range [IQR]) and categorical variables as number (%). Eye-level AL slopes were analyzed using a rank-based linear mixed-effects model, with age decade as the fixed effect and participant as the random intercept. Pairwise decade comparisons were adjusted using the Holm–Bonferroni method. Baseline group comparisons were performed for descriptive and exploratory purposes using the Kruskal–Wallis test for continuous variables and the chi-square test for categorical variables; *P* values were adjusted for multiple comparisons using Holm–Bonferroni method. Two-sided *P* values <0.05 were considered statistically significant.

To address outcome imbalance across severity categories (with nil or mild class predominating and severe class being rare), we implemented inverse class-frequency weighting, applying class weights defined as *w*_*k*_ *= N*_*total*_ */ (K·N*_*k*_*)*. The ordered severity categories (nil/mild/moderate/severe) served as the dependent variable in a class-weighted proportional-odds ordinal logistic regression. Intereye correlation was addressed by computing cluster-robust (Huber–White) standard errors with participant ID as the clustering unit, which yields valid inference under arbitrary within-person correlation while leaving point estimates unchanged. Candidate variables identified from the baseline group comparisons were considered for entry, and the final set of predictors was determined by Akaike information criterion–guided stepwise selection. Multicollinearity was considered acceptable at a variance inflation factor or generalized variance inflation factor was <5. Because SER is strongly correlated with AL, AL and questionnaire-based cataract surgery history were included as candidate covariates, and SER was prespecified for exclusion from multivariable modeling. Likewise, because hematocrit is strongly correlated with hemoglobin and red blood cell count, hematocrit was prespecified for exclusion. Results were reported as odds ratios (ORs) with 95% confidence intervals (CIs).

To determine which eye elongated faster within each participant, we defined a pair-level binary outcome equal to 1 if the baseline longer eye’s AL slope exceeded that of the fellow eye and 0 otherwise. Pairs without interocular AL asymmetry (baseline interocular difference = 0 mm) and ties (absolute between-eye difference in slopes within a numerical tolerance) were excluded. We then fitted a single change-point logistic regression with the longer eye’s baseline AL as the predictor and the interocular difference in AL as a linear covariate. The change-point location was selected by Akaike information criterion over a 24.0 to 29.0 mm grid at 0.05-mm increments. The AL value at which the predicted probability equals 0.50 was obtained by numerical root-finding with the interocular AL difference fixed at the sample median. Uncertainty for the change-point and the 0.50 crossing was quantified by percentile bootstrap with 500 participant-level resamples. For visualization, a 1.0-mm running-window estimate with 0.10-mm steps was overlaid on the model-based curve.

In additional analyses, to assess potential nonlinearity between age and the cumulative probability of at least mild axial elongation, we fitted proportional-odds models with restricted cubic splines. Knot locations were determined from the age distribution. We estimated the age corresponding to the minimum predicted risk and used this value as a cutoff for stratified analyses; within each age stratum, the same proportional-odds modeling framework was applied.

## Results

A total of 4016 participants (8032 eyes) met the criteria and were included in the analysis, contributing 21 421 visits. The median number of visits was 6 (IQR, 4–7), and the median follow-up duration was 5.31 years (IQR, 3.83–6.01 years). Baseline characteristics are summarized in Table 1. At baseline, 70.5% met the myopia threshold (SER less than or equal to –0.50 diopters [D]; 5665 eyes) and 10.3% met the high-myopia threshold (SER less than or equal to –6.00 D; 830 eyes); for AL, 17.8% of eyes had AL ≥26.0 mm (1426 eyes) and 2.0% had AL ≥28.0 mm (162 eyes).

**Table 1:** Baseline Characteristics Stratified by Severity of Annual Axial Elongation

Characteristic	Nil (<0 mm/yr)	Mild (≥0 and <0.05 mm/yr)	Moderate (≥0.05 and <0.10 mm/yr)	Severe (≥0.10 mm/yr)	Total	*P* Value
Eyes, n (%)	4077 (50.8%)	3836 (47.8%)	105 (1.3%)	14 (0.2%)	8032	–
Right eyes, n (%)	2054 (50.4%)	1904 (49.6%)	49 (46.7%)	9 (64.3%)	4016 (50.0%)	0.562
AL growth (mm/year)	–0.01 (–0.02 to –0.00) [–0.15 to –0.00]	0.01 (0.00–0.02) [0.00–0.05]	0.06 (0.06–0.07) [0.05–0.10]	0.11 (0.11–0.13) [0.10–0.20]	–0.00 (–0.01 to 0.01) [–0.15 to 0.20]	–
AL (mm)	24.45 (23.60–25.51) [20.94–29.84]	24.43 (23.55–25.57) [20.80–31.79]	25.87 (24.63–27.53) [22.14–32.70]	27.69 (26.06–29.29) [23.24–31.82]	24.45 (23.57–25.57) [20.80–32.70]	<0.001∗∗
Interocular difference in AL (mm)	0.15 (0.07–0.29) [0.00–2.43]	0.15 (0.07–0.27) [0.00–2.67]	0.22 (0.10–0.49) [0.00–2.67]	0.70 (0.58–0.80) [0.17–1.70]	0.15 (0.07–0.28) [0.00–2.67]	<0.001∗∗
CVA (LogMAR units)	0.00 (–0.08 to 0.10) [–0.18 to 1.00]	0.00 (–0.08 to 0.10) [–0.18 to 1.00]	0.05 (0.00–0.22) [–0.18 to 1.00]	0.10 (0.01–0.26) [–0.18 to 0.40]	0.00 (–0.08 to 0.10) [–0.18 to 1.00]	<0.001∗∗
SER	–1.50 (–3.62 to –0.25) [–13.25 to 6.88]	–1.50 (–4.00 to –0.12) [–13.38 to 5.75]	–4.50 (–7.25 to –1.50) [–15.00 to 3.75]	–5.38 (–8.78 to –4.12) [–14.62 to 1.00]	–1.50 (–3.88 to –0.25) [–15.00 to 6.88]	<0.001∗∗
IOP (mmHg)	12.00 (10.00–15.00) [7.00–31.00]	12.00 (10.00–14.00) [7.00–25.00]	12.00 (10.00–14.00) [8.00–20.00]	14.50 (13.25–15.00) [8.00–19.00]	12.00 (10.00–14.00) [7.00–31.00]	0.986
History of cataract surgery, n (%)	232 (5.7%)	223 (5.8%)	15 (14.3%)	2 (14.3%)	472 (5.9%)	0.001∗
Glaucoma treatment, n (%)	183 (4.5%)	176 (4.6%)	8 (7.6%)	3 (21.4%)	370 (4.6%)	0.010∗
META-PM category (0/1/2/3/4)	4034/40/2/0/1	3766/61/4/3/2	85/15/3/2/0	8/3/1/2/0	7893/119/10/7/3	<0.001∗∗
Age (yrs)	53.38 (45.93–61.27) [24.03–92.96]	54.23 (46.41–62.38) [22.38–88.67]	50.62 (42.53–60.35) [22.38–80.38]	49.15 (42.42–51.44) [39.94–78.60]	53.89 (46.08–61.77) [22.38–92.96]	0.005∗
Body height (cm)	167.30 (160.80–172.80) [137.70–187.40]	166.50 (159.70–172.00) [137.20–191.60]	165.60 (159.80–171.30) [147.30–185.60]	164.50 (158.40–172.88) [154.30–187.30]	166.85 (160.20–172.40) [137.20–191.60]	0.027∗
Weight (kg)	65.30 (56.60–73.30) [32.10–139.80]	64.40 (56.20–72.70) [26.90–139.80]	62.30 (54.40–69.00) [39.40–102.70]	66.45 (48.15–74.80) [42.40–89.20]	64.90 (56.30–73.00) [26.90–139.80]	0.080
BMI (kg/m^2^)	23.20 (21.00–25.50) [14.20–47.60]	23.10 (21.00–25.60) [12.10–47.60]	22.90 (20.10–25.30) [16.10–33.70]	22.95 (18.85–25.45) [17.60–27.10]	23.20 (21.00–25.50) [12.10–47.60]	1.000
Female, n (%)	1147 (28.1%)	1256 (32.7%)	46 (43.8%)	7 (50.0%)	2456 (30.6%)	<0.001∗∗
sBP (mmHg)	115 (104–125) [72–197]	114 (104–126) [78–213]	111 (99–122) [77–153]	106 (99–111) [92–163]	114 (104–125) [72–213]	0.609
dBP (mmHg)	74 (66–81) [37–123]	73 (65–80) [38–137]	69 (62–77) [48–108]	68 (65–82) [53–98]	73 (66–81) [37–137]	0.004∗
Pulse rate (per min)	68 (61–75) [31–132]	67 (61–75) [34–115]	67 (64–74) [53–120]	67 (64–70) [50–95]	68 (61–75) [31–132]	1.000
WBC (×10^3^/μL)	5.00 (4.30–6.00) [1.90–14.00]	5.00 (4.20–6.00) [1.90–14.60]	4.80 (4.20–5.80) [3.00–8.60]	4.45 (3.58–4.95) [3.00–6.00]	5.00 (4.20–6.00) [1.90–14.60]	0.700
RBC (×10^6^/μL)	4.74 (4.45–5.05) [3.33–6.38]	471 (440–502) [33–637]	471 (434–49) [375–602]	455 (42–503) [411–543]	472 (442–503) [333–638]	0.090
Hb (g/dL)	14.80 (13.80–15.50) [6.30–19.30]	14.60 (13.70–15.50) [6.30–19.30]	14.30 (13.50–15.50) [11.50–18.20]	13.80 (13.18–15.50) [11.70–16.70]	14.70 (13.70–15.50) [6.30–19.30]	0.024∗
Ht (%)	43.30 (41.10–45.50) [25.40–59.10]	43.00 (40.50–45.30) [25.90–59.10]	42.40 (40.00–45.10) [34.70–52.40]	39.80 (39.40–45.45) [34.40–47.90]	43.20 (40.80–45.40) [25.40–59.10]	0.002∗
Plt (×10^3^/μL)	232 (198–266) [74–724]	230 (198–263) [74–500]	223 (202–258) [134–368]	211 (198–239) [169–304]	230 (198–265) [74–724]	1.000
TG (mg/dL)	97.00 (68.00–141.00) [21.00–2946.00]	97.00 (68.00–141.00) [18.00–1520.00]	93.00 (65.00–145.00) [33.00–520.00]	96.50 (72.00–141.50) [37.00–205.00]	97.00 (68.00–141.00) [18.00–2946.00]	1.000
HDL (mg/dL)	58.00 (50.00–70.00) [22.00–127.00]	59.00 (50.00–70.25) [25.00–127.00]	60.00 (50.00–69.00) [33.00–115.00]	55.00 (54.25–60.75) [47.00–79.00]	59.00 (50.00–70.00) [22.00–127.00]	1.000
LDL (mg/dL)	127.00 (108.00–148.00) [48.00–266.00]	126.00 (107.00–146.00) [25.00–317.00]	119.00 (100.00–144.00) [72.00–177.00]	126.00 (104.25–141.75) [77.00–157.00]	126.00 (107.00–147.00) [25.00–317.00]	0.797
Total cholesterol (mg/dL)	202.00 (180.00–224.00) [106.00–350.00]	202.00 (180.00–225.00) [106.00–400.00]	195.00 (178.00–217.00) [141.00–284.00]	199.50 (181.75–217.00) [133.00–239.00]	202.00 (180.00–224.00) [106.00–400.00]	1.000
FBS (mg/dL)	98.00 (92.00–105.00) [60.00–439.00]	97.00 (92.00–105.00) [60.00–350.00]	95.00 (89.00–102.00) [77.00–153.00]	94.00 (89.25–99.75) [81.00–118.00]	98.00 (92.00–105.00) [60.00–439.00]	0.393
HbA1c (%)	5.60 (5.40–5.90) [4.20–14.30]	5.60 (5.40–5.90) [4.40–13.80]	5.60 (5.40–5.70) [4.50–7.10]	5.60 (5.43–5.78) [5.20–6.10]	5.60 (5.40–5.90) [4.20–14.30]	0.235
Cre (mg/dL)	0.78 (0.67–0.89) [0.35–2.37]	0.77 (0.66–0.88) [0.36–1.92]	0.75 (0.64–0.86) [0.44–1.16]	0.74 (0.62–0.86) [0.51–0.93]	0.78 (0.67–0.88) [0.35–2.37]	0.329
eGFR (mL/min/1.73 m^2^)	76.60 (68.10–85.80) [25.20–151.20	76.10 (67.30–85.70) [28.20–142.60]	77.60 (68.50–88.10) [50.50–126.90]	78.35 (70.85–88.45) [60.20–104.60]	76.40 (67.80–85.80) [25.20–151.20]	1.000
AST (U/L)	21 (18–26) [9–191]	21 (18–25) [7–225]	21 (16–27) [10–63]	19 (16–23) [10–28]	21 (18–26) [7–225]	1.000
ALT (U/L)	20 (14–28) [3–196]	19 (14–28) [3–163	20 (13–31) [5–160]	17 (11–20) [5–33]	19 (14–28) [3–196]	1.000
γ-GTP (U/L)	28 (18–48) [5–452]	27 (17–46) [5–535]	24 (16–41) [8–322]	18 (12–31) [7–157]	27 (18–46) [5–535]	0.077
ALP (U/L)	187 (152–226) [27–707]	187 (150–227) [29–607]	194 (149–236) [40–379]	152 (125–194) [70–264]	187 (15–22) [27–707]	1.000
Antihypertensive medication use, n (%)	722 (17.7%)	731 (19.1%)	21 (20.0%)	2 (14.3%)	1476 (18.4%)	0.435
Antidiabetic medication use, n (%)	243 (6.0%)	210 (5.5%)	7 (6.7%)	0 (0.0%)	460 (5.7%)	0.596
Lipid-lowering medication use, n (%)	533 (13.1%)	533 (13.9%)	20 (19.0%)	2 (14.3%)	1088 (13.5%)	0.273
Habitual exercise, n (%)	976 (23.9%)	886 (23.1%)	16 (15.2%)	4 (28.6%)	1882 (23.4%)	0.175
Habitual walking, n (%)	842 (20.7%)	803 (20.9%)	19 (18.1%)	0 (0.0%)	1664 (20.7%)	0.239
History of alcohol intake, n (%)	2792 (68.5%)	2559 (66.7%)	58 (55.2%)	9 (64.3%)	5418 (67.5%)	0.017∗
History of smoking, n (%)	2124 (52.1%)	1964 (51.2%)	47 (44.8%)	7 (50.0%)	4142 (51.6%)	0.453

The severity of axial elongation was classified as follows: (1) severe, ≥0.10 mm/year; (2) moderate, ≥0.05 and <0.10 mm/year; (3) mild, ≥0 and <0.05 mm/year; and (4) nil, <0 mm/year. Data are presented as median (interquartile range: 25th percentile–75th percentile) [range] for continuous variables and number (percentage) for categorical variables. *P* values were obtained using Kruskal–Wallis tests for continuous variables and χ^2^ or Fisher exact tests for categorical variables, and were adjusted for multiple comparisons using the Holm–Bonferroni adjustment.

AL = axial length; ALP = alkaline phosphatase; ALT = alanine aminotransferase; AST = aspartate aminotransferase; BMI = body mass index; Cre = creatinine; CVA = corrected visual acuity; dBP = diastolic blood pressure; eGFR = estimated glomerular filtration rate; FBS = fasting blood sugar; Hb = hemoglobin; HbA1c = hemoglobin A1c; HDL = high-density lipoprotein cholesterol; Ht = hematocrit; IOP = intraocular pressure; LDL = low-density lipoprotein cholesterol; LogMAR = logarithm of the minimum angle of resolution; META-PM = Meta-Analysis for Pathologic Myopia; Plt = platelet; RBC = red blood cell count; sBP = systolic blood pressure; SER = spherical equivalent refraction; TG = triglyceride; WBC = white blood cell count; γ-GTP = γ-glutamyl transpeptidase.

Significant values are denoted as follows: ∗*P* < 0.05; ∗∗*P* < 0.001.

**Figure 1:**
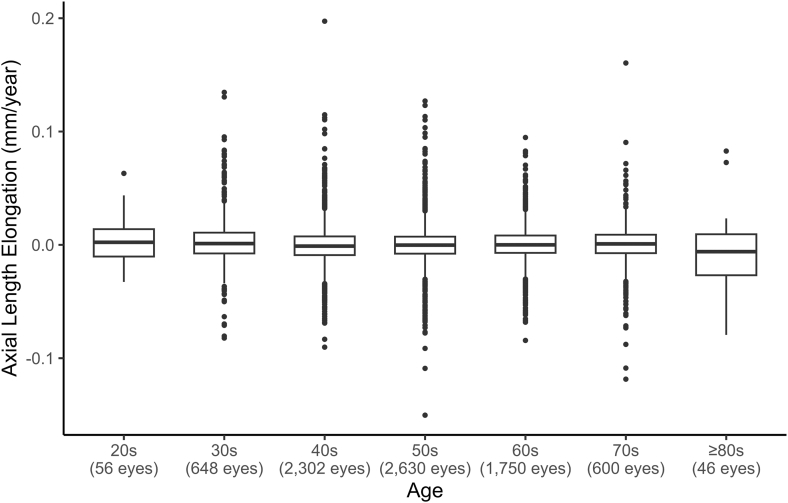
Box plots of annual axial elongation by age group. Boxes represent the median and interquartile range. Medians of the rate of axial length elongation were approximately 0 across decades.

Participant ages at baseline ranged from 22.4 to 93.0 years. The distribution of participants by decade was 0.7% in their 20s (n = 28), 8.1% in their 30s (n = 324), 28.7% in their 40s (n = 1151), 32.7% in their 50s (n = 1315), 21.8% in their 60s (n = 875), 7.5% in their 70s (n = 300), and 0.6% at ≥80 years (n = 23), indicating that this cohort was predominantly middle-aged. Annual axial elongation was approximately centered on zero across decades (Fig 1): decade-specific medians ranged from –0.01 to 0.00 mm/year at both the eye- and participant-levels (Table S1, available at www.ophthalmologyscience.org). In a rank-based linear mixed-effects model with a participant-level random intercept, decade showed a small but statistically significant overall effect (*P* = 0.020). In post hoc comparisons using estimated marginal means with Holm–Bonferroni adjustment, the 30s group had slightly higher ranks than the 40s group (adjusted *P* = 0.038).

### Distribution of Annual Axial Elongation by Severity

Based on the per-eye annual axial elongation rate, eyes were distributed across categories as follows: 4077 (50.8%), 3836 (47.8%), 105 (1.3%), and 14 (0.2%) for nil, mild, moderate, and severe, respectively. The median annual elongation rate (mm/year) for these categories was –0.01 (IQR –0.02 to –0.00), 0.01 (IQR 0.00–0.02), 0.06 (IQR 0.06–0.07), and 0.11 (IQR 0.11–0.13); baseline clinical features stratified by severity are summarized in Table 1. Between-group differences were significant for baseline AL, interocular difference in AL, corrected visual acuity, SER, META-PM classification, age, female sex, height, diastolic blood pressure, hemoglobin, hematocrit, history of cataract surgery, glaucoma treatment, and history of alcohol intake (all *P* < 0.05 after Holm–Bonferroni adjustment; Table 1).

### Factors Associated with Severity of Annual Axial Elongation

**Figure 2:**
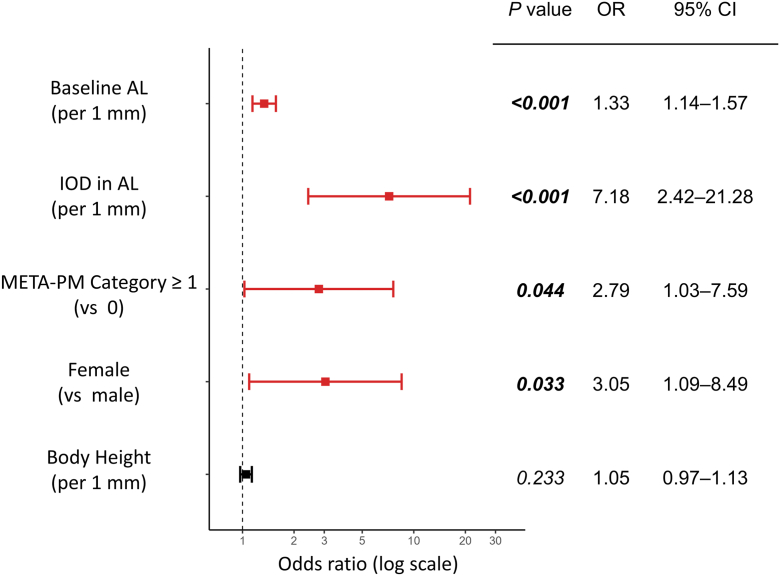
Factors associated with greater severity of annual axial elongation. Odds ratios with 95% CIs from a class‑weighted proportional‑odds model with cluster‑robust standard errors for severity of annual axial elongation (nil/mild/moderate/severe). Baseline AL, IOD in AL, META-PM category (≥1), and female sex were identified as independent factors (all *P* < 0.05), whereas body height was not associated after adjustment (*P* = 0.233). AL = axial length; CI = confidence interval; IOD = interocular difference; META-PM = Meta‑Analysis for Pathologic Myopia; OR = odds ratio.

In the class-weighted proportional-odds model of severity (nil/mild/moderate/severe), the following factors were independently associated with greater severity (Fig 2): baseline AL (per 1 mm increase), the OR was 1.34 (95% CI, 1.14–1.57; *P* < 0.001); interocular difference in AL (per 1 mm increase), the OR was 7.18 (2.42–21.28; *P* < 0.001); META-PM category ≥1 (vs. 0), the OR was 2.79 (1.03–7.59; *P* = 0.044); and female sex (vs. male), the OR was 3.05 (1.09–8.49; *P* = 0.033). Height (per 1 cm increase) was not significant after adjustment (OR, 1.05; 95% CI, 0.97–1.13; *P* = 0.233).

### Probability of Faster Elongation in the Longer Eye Stratified by Baseline Axial Length

**Figure 3:**
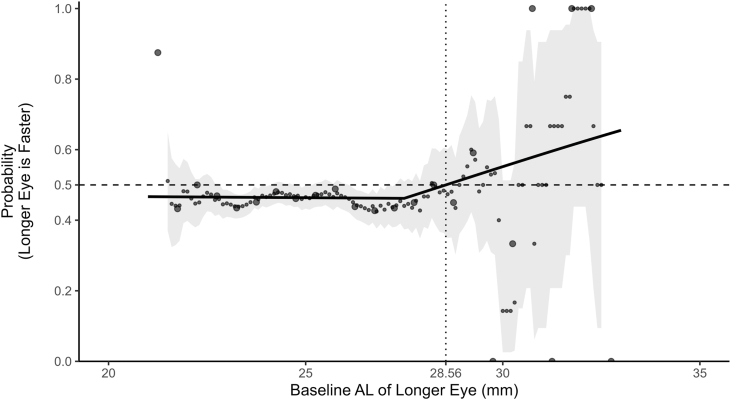
Relationship between baseline AL and the probability that the longer eye elongates faster. The curve shows predictions from a segmented logistic regression with a single change-point (27.50 mm, chosen by Akaike information criterion) and a linear effect of interocular difference in AL. For display, interocular difference in AL is held at the median (0.15 mm). The horizontal line marks 0.50; the vertical line marks the model‑estimated crossing at 28.56 mm (bootstrap median 28.62 mm; 95% CI, 27.50–30.08; 500 participant‑level bootstrap resamples). Points show a 1.0‑mm running‑window estimate (step 0.10 mm) overlaid on the model; wider uncertainty at very large AL reflects sparse data. AL = axial length; CI = confidence interval.

To determine whether the baseline longer eye elongated faster within each pair with interocular AL asymmetry, we derived a pair-level binary outcome equal to 1 if the longer eye’s AL slope exceeded that of the fellow eye and 0 otherwise. Ninety-one pairs with interocular difference in AL equaling 0 mm (no longer-eye designation) and 29 pairs with identical intereye slopes (indeterminate outcome) were excluded, leaving 3896 pairs (7792 eyes) for analysis. We modeled the relationship using a segmented (single change-point) logistic regression with interocular difference in AL as a linear covariate; the Akaike information criterion selected a change-point at 27.50 mm. With interocular difference in AL fixed at its median (0.15 mm), the predicted probability that the longer eye elongated faster crossed 0.50 at 28.56 mm in the longer eye (bootstrap median 28.62 mm; 95% CI, 27.50–30.08; 500 participant-level resamples); below this, estimates were about 0.50 (no consistent lateral preference), and above this value, they exceeded 0.50 with wider uncertainty at extreme AL (Fig 3). A 1.0-mm running-window display (step 0.10 mm) overlaid on the model reproduced the same pattern—essentially flat below 27.5 mm and rising beyond 28.5–29.0 mm—while highlighting greater imprecision at very large AL.

### Stratified Analyses by Age Cutoff

**Figure 4:**
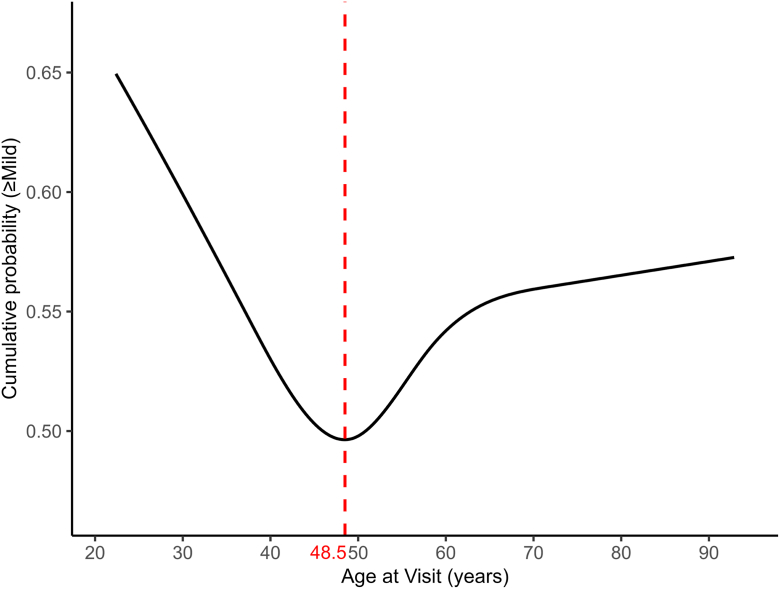
Nonlinear association between age and the cumulative probability of at least mild axial elongation. The model‑predicted cumulative probability that an eye exhibits at least mild axial elongation (≥0 mm/year; i.e., mild, moderate, or severe vs. nil) is shown as a function of age. Predictions were obtained from a proportional‑odds ordinal logistic regression with age modeled using a restricted cubic spline; knot locations were chosen from the empirical age distribution. The curve displays a convex pattern with a data‑driven nadir at 48.5 years, indicating lower risk near mid‑life and higher probabilities at both younger and older ages relative to the nadir. The red vertical line marks the minimum at 48.5 years.

In proportional-odds models with restricted cubic splines, the association between age and the cumulative probability of at least mild axial elongation was nonlinear, with a minimum around 48.5 years (Fig 4). Using this data-driven cutoff, we stratified participants into <48.5 years and ≥48.5 years. In both age strata, baseline AL and interocular difference in AL were independently associated with greater severity: for baseline AL (per 1 mm), the OR was 1.68 (95% CI, 1.35–2.08) and for interocular difference in AL (per 1 mm), the OR was 11.90 (3.02–46.94) in those <48.5 years and 1.43 (1.12–1.82) and 4.90 (1.08–22.24) in those ≥48.5 years. Additionally, in the <48.5-year group, serum creatinine showed a significant inverse association, as a protective effect (OR, 0.05; 95% CI, 0.004–0.60; *P* = 0.019), whereas in the ≥48.5-year group, sex and hemoglobin were retained in the model but were not significantly associated (all *P* > 0.05; Table S2, available at www.ophthalmologyscience.org).

## Discussion

In this longitudinal, population-based Japanese cohort (n = 4016; median follow-up 5.31 years), AL elongation in adults was generally uncommon and slow. However, readily measured features—longer baseline AL (AL ≥ 28.0 mm), greater interocular difference in AL (interocular difference in AL ≥ 0.30 mm), presence of early myopic macular change (META-PM ≥1), and female sex—delineated a higher-risk phenotype. In addition, at the pair level, no consistent lateral preference was evident below approximately 28.6 mm; above this intersection, the longer eye was more likely to elongate faster, with wider uncertainty at extreme AL. These descriptive estimates may inform follow-up decisions in health-check settings.

### Determinants of Severe Elongation

Using a class-weighted proportional-odds model with cluster-robust standard errors to address outcome imbalance and intereye correlation, we found independent associations with greater severity of AL elongation for baseline AL (OR, 1.34 per 1 mm), interocular difference in AL (OR, 7.18 per 1 mm), META-PM ≥1 (OR, 2.79), and female sex (OR, 3.05). Notably, a retrospective cohort of young adults with nonpathologic high myopia reported the same directions of association—baseline AL (particularly at ≥28 mm), interocular axial asymmetry, and female sex—as independent determinants of more severe elongation.^7^ In line with that study, the approximately 28-mm baseline AL threshold is consistent with the pair-level intersection at 28.56 mm in our analysis, reinforcing its clinical relevance across distinct populations. These results also extend hospital- or clinic-based observations to a general screening population and indicate that ocular measurements available at routine visits may be effective for stratifying adult risk.

In this study, baseline myopic maculopathy graded by the META-PM classification was operationalized as an ocular structural phenotype that captures posterior-segment remodeling and biomechanical vulnerability not fully reflected by AL. This specification was based on adult evidence showing that retinal features including posterior staphyloma characterize globes prone to further axial elongation,^3^^,^^9^ thereby conveying local susceptibility beyond AL alone. To enhance comparability across studies and clinical strata, the standardized, reproducible META-PM system^8^^,^^10^ was adopted. In particular, META-PM ≥1 was used as an index of early myopic macular change—including tessellated fundus—rather than as pathologic myopia per se, thereby indicating local susceptibility along a continuum before overt atrophy develops. Clinically, the observed positive association between META-PM ≥1 and more-severe elongation in this cohort indicates that baseline retinal structural change does not imply universal cessation of axial elongation; risk-stratified surveillance remains warranted even in eyes with tessellation or more advanced myopic maculopathy.

Prior evidence indicates that female sex is associated with a greater myopia burden across study designs; population-based cross-sectional studies in Japan report a higher prevalence of high myopia among women^11^^,^^12^ and a prospective cohort study has reported similar patterns, with higher incidence and faster axial elongation in women.^3^ However, adult women typically have shorter AL^13^^,^^14^; therefore, analyses that rely on high-myopia thresholds (e.g., AL ≥26 mm) can under-identify at-risk women who nonetheless continue to elongate. Conversely, threshold-restricted designs may also overstate associations in women by preferentially sampling those with extreme AL and faster elongation. In our population-based analysis, which did not restrict enrollment by AL, female sex remained independently associated with more severe AL elongation (OR, 3.05; 95% CI, 1.09–8.49). Importantly, a recent Japanese adult cohort—not restricted to high myopia—also found a higher risk of axial elongation in women.^15^ Because that cohort was female-predominant (73.3% female), representing the opposite of our workplace-based health-check sample (30.6% female), this convergence reduces the likelihood that the female-sex signal is an artifact of sex composition. Potential contributors include behavioral patterns (e.g., greater screen use and indoor activity).^16^ However, the CI around the female-sex effect was wide and, in age-stratified models, sex was not consistently significant; therefore, absolute-risk calibration may still vary by sex distribution and setting. Accordingly, these findings should be interpreted with caution to avoid overgeneralization.

### Interocular Dynamics across Baseline Axial Length

Pair-level change-point modeling suggested that any lateral preference in axial elongation emerges only at high baseline AL values. With interocular difference in AL fixed at the cohort median (0.15 mm), the predicted probability that the longer eye elongates faster intersected 0.50 at 28.56 mm (bootstrap 95% CI, 27.50–30.08). Below 28.56 mm, estimates hovered around 0.50—that is, no consistent preference for either eye; beyond 28.56 mm, the longer eye became more likely to elongate faster, with uncertainty increasing at extreme AL because of sparse data. Although prior reports seem divergent,^7^^,^^17^ the discrepancy between a large longitudinal cohort of young adults with nonpathologic high myopia (no significant difference)^7^ and a comparison study of unilateral high myopia in adults (longer-eye–predominant)^17^ may be explained by where each cohort lies relative to the AL range around this intersection. Rather than a prescriptive rule, this single curve may help organize expectations: populations distributed predominantly to the left of the intersection (≤28.56 mm) would be expected to show no consistent lateral preference,^7^ whereas those to the right (≥28.56 mm) may more often show longer-eye–predominant elongation,^17^ with uncertainty increasing at extreme AL. By examining a broad AL range that extends beyond classical high myopia, the present study enables this unified interpretation. Mechanistically, the pattern is consistent with a shift from visually guided emmetropization at shorter ALs^18^ toward biomechanical vulnerability in highly elongated globes.^19^^,^^20^

### Myopia Progression in Young Adults

Several reports implicate younger adult age as a risk factor for myopia progression, particularly among highly myopic individuals. A prior report in nonpathologic high myopia identified younger age as a determinant of more-severe axial elongation.^7^ Recent syntheses indicated that refractive progression can persist into early adulthood (20–30 years) and is faster with greater baseline myopia.^21^^,^^22^ Population-based and electronic medical record studies likewise reported that most adults remain stable, whereas a subset of younger adults and those with high myopia exhibit clinically relevant progression.^23^ Conversely, in cohorts incorporating detailed environmental and genetic profiling, the average magnitude of axial elongation during young adulthood was small and the variance explained by screen exposure, ocular sun exposure, and polygenic risk was limited.^24^ In our population-based health-check cohort, exploratory spline modeling revealed a nonlinear pattern in which the predicted probability of at least mild elongation (≥0 mm/year) showed a shallow nadir around midlife (48.5 years). However, decade-specific medians of annual axial elongation remained consistently near zero (–0.01 to 0.00 mm/year) across all age groups, including participants in their 20s and 30s. Multivariable models identified ocular structural characteristics—longer baseline AL, greater interocular asymmetry, and early myopic macular changes—as independent determinants of more-severe elongation, whereas age contributed only limited independent information once these factors were considered. These findings suggest that risk concentrates in structurally vulnerable eyes and that age may act primarily as a surrogate for residual elongation potential in highly myopic eyes. Nevertheless, because this was a predominantly middle-aged cohort (0.7% in their 20s; 8.1% in their 30s), the sample of young adults may have been insufficient to fully detect age-specific elongation risk, and generalization to early adulthood should be made with caution.

### Mechanistic Context and Nonassociations

After accounting for baseline AL and interocular difference in AL, our results indicate that additional adult axial elongation is governed primarily by local ocular biomechanics and choroidal or scleral remodeling, rather than by systemic lipid status acting as an independent driver. For anthropometric and metabolic factors, prior literature has reported inconsistent associations between body mass index or anthropometry and AL—with large population-based studies finding positive associations^25^ and inverse associations.^26^ Against this backdrop, we explicitly included blood assays related to lipid metabolism (e.g., cholesterol fractions), yet none emerged as independent determinants of adult AL elongation. These observations do not support the hypothesis that lipid status directly drives additional elongation in adulthood; rather, they suggest that control of adult elongation is primarily ocular-local, particularly scleral biomechanics and remodeling, with only a minor contribution from systemic lipid metabolism—or effects already captured by baseline AL or other ocular factors.^3^^,^^4^^,^^20^^,^^27^, ^28^, ^29^, ^30^

One nonlipid biochemical showed an age-limited signal: among adults <48.5 years, higher serum creatinine was associated with lower odds of more severe elongation, consistent with a modest protective effect, whereas no association was observed in those ≥48.5 years. Notably, female sex was not retained in the multivariable model in the younger stratum, whereas the female sex term was retained and creatinine did not in the older stratum. Variance inflation factors were low in both strata, arguing against multicollinearity and instead suggesting age-specific proxy substitution in what these variables capture—creatinine acting as a body-composition proxy (partly reflecting sex-related differences in muscle mass) in younger adults, with female sex representing this information more directly in older adults. This interpretation accords with well-documented sex differences in serum creatinine across populations and with creatinine’s dependence on muscle mass; minor elevations in muscular or supplement-using individuals can be physiologic rather than renal injury.^31^

### Strengths and Limitations

This study has several strengths: first, it draws on a large, population-based cohort of 4016 adults; second, it estimates per-eye annual AL change rigorously using within-person regressions across multiple time points with a median follow-up exceeding 5 years; third, it simultaneously addresses intereye correlation and outcome class imbalance via participant-level clustering and inverse class-frequency weighting. This study also has several limitations. First, the severe category included only 14 eyes (0.2%), resulting in wide CIs and warranting caution in recommendations specific to this group; confirmation in larger cohorts is needed. Second, variable selection and availability may limit generalizability and causal inference: educational attainment,^25^^,^^32^, ^33^, ^34^ which has been associated with AL, was not captured in the questionnaire; and the cohort was restricted to individuals who elected ophthalmic testing within the health-check program. In addition, participation did not follow a probability sampling scheme; the program’s substantial workplace-based component resulted in an overrepresentation of men (69.4% men; 30.6% women), potentially yielding a relatively homogeneous profile and limiting generalizability. Third, refractive error was measured with an autorefractor without cycloplegia; although this does not affect AL measurement, residual accommodation may bias SER. Therefore, SER-based descriptors should be interpreted with caution. Finally, given the observational design, residual confounding cannot be fully excluded, and the results should be interpreted as associations rather than causal effects.

### Conclusions

This study indicates that although adult AL elongation is rare and slow, higher risk concentrates among eyes with longer AL, greater interocular difference in AL, early myopic maculopathy including tessellated fundus, and in women; these readily measured features may inform follow-up planning in health-check settings. Pair-level estimates around 28.6 mm may also help prioritize eyes for follow-up but should be interpreted with caution. The rarity of severe cases leaves imprecision in estimates, underscoring the need for further validation to improve precision and generalizability.
