# Supplementary material for: Risk Factors for Adult Axial Length Elongation: A 5-Year Population-Based Cohort Study
Source: Ophthalmol Sci. 2025 Nov 17;6(2):101011. doi: 10.1016/j.xops.2025.101011 (PMC12794474; doi:10.1016/j.xops.2025.101011)
Supplement: Supplementary_Table S1 [file mmc1.pdf]

Table S1. Annualized axial length change by age decade at the eye- and participant-levels

Decade	Eyes, n (%)	AL growth per eye (mm/year)	Participants, n (%)	AL growth per perticipant (mm/year)
20s	56 (0.7%)	0.00 (−0.01, 0.01) [−0.03, 0.06]	28 (0.7%)	0.00 (−0.01, 0.01) [−0.02, 0.04]
30s	648 (8.1%)	0.00 (−0.01, 0.01) [−0.08, 0.13]	324 (8.1%)	0.00 (−0.01, 0.01) [−0.08, 0.13]
40s	2,302 (28.7%)	0.00 (−0.01, 0.01) [−0.09, 0.20]	1,151 (28.7%)	0.00 (−0.01, 0.01) [−0.07, 0.09]
50s	2,630 (32.7%)	0.00 (−0.01, 0.01) [−0.15, 0.13]	1,315 (32.7%)	0.00 (−0.01, 0.01) [−0.13, 0.12]
60s	1,750 (21.8%)	0.00 (−0.01, 0.01) [−0.08, 0.09]	875 (21.8%)	0.00 (−0.01, 0.01) [−0.06, 0.08]
70s	600 (7.5%)	0.00 (−0.01, 0.01) [−0.12, 0.16]	300 (7.5%)	0.00 (−0.01, 0.01) [−0.11, 0.08]
≥ 80s	46 (0.6%)	−0.01 (−0.03, 0.01) [−0.08, 0.08]	23 (0.6%)	−0.01 (−0.02, 0.01) [−0.07, 0.08]

AL = axial length.

Data are presented as median (interquartile range: 25th percentile–75th percentile) [range] for continuous variables and number (percentage) for categorical variables.

Eye-level values were computed using all eyes independently, whereas participant-level values were based on the mean of both eyes for each participant.
