# Supplementary material for: Risk Factors for Adult Axial Length Elongation: A 5-Year Population-Based Cohort Study
Source: Ophthalmol Sci. 2025 Nov 17;6(2):101011. doi: 10.1016/j.xops.2025.101011 (PMC12794474; doi:10.1016/j.xops.2025.101011)
Supplement: Supplementary_Table S2 [file mmc2.pdf]

Table S2. Multivariable proportional-odds models stratified by age

Group	Parameter	OR [95% CI]	*P* value	VIF
<48.5 years	AL (mm)	1.68 [1.35, 2.08]	<0.001**	1.09
	Interocular difference in AL (mm)	11.90 [3.02, 46.9]	<0.001**	1.04
	Creatinine (mg/dL)	0.05 [0.04, 0.60]	0.019*	1.06
≥48.5 years	AL (mm)	1.43 [1.12, 1.82]	0.004*	1.57
	Interocular difference in AL (mm)	4.90 [1.08, 22.24]	0.040*	1.57
	Sex (Male:0, Female:1)	1.97 [0.64, 6.08]	0.236	1.58
	Hb (g/dL)	1.26 [0.77, 2.07]	0.359	1.64

AL = axial length; CI = confidence interval; Cre = creatinine; Hb = hemoglobin; VIF = variance inflation factor.

**P* <0.05; ***P* <0.001.

Odds ratios are per 1-mm increase in AL and interocular difference in AL, per 1-mg/dL increase in creatinine, and per 1-g/dL increase in hemoglobin.
